# Right bundle branch block pattern with right ventricular pacing in arrhythmogenic right ventricular cardiomyopathy

**DOI:** 10.1007/s10840-025-02051-6

**Published:** 2025-04-22

**Authors:** Adrian M. Petzl, Michael I. Gurin, Francis E. Marchlinski

**Affiliations:** Electrophysiology Section, Division of Cardiovascular Medicine, Department of Medicine, Hospital of the University of Pennsylvania, Philadelphia, PA USA

Arrhythmogenic right ventricular cardiomyopathy (ARVC) is characterized by a progressive fibrofatty replacement of the right ventricular myocardium [1]. This often leads to severe right ventricular (RV) dilatation and delayed electrical activation. According to the 2019 HRS expert consensus statement on ARVC, only left bundle branch block morphology ventricular tachycardia (VT) constitute major and minor diagnostic criteria [2]. However, in patients with ARVC, RV apical pacing and reentrant VT exiting from the RV apical region may produce an unexpected right bundle branch block (RBBB) pattern due to altered anatomical relationships and conduction delays [3, 4].

**Fig. 1:**
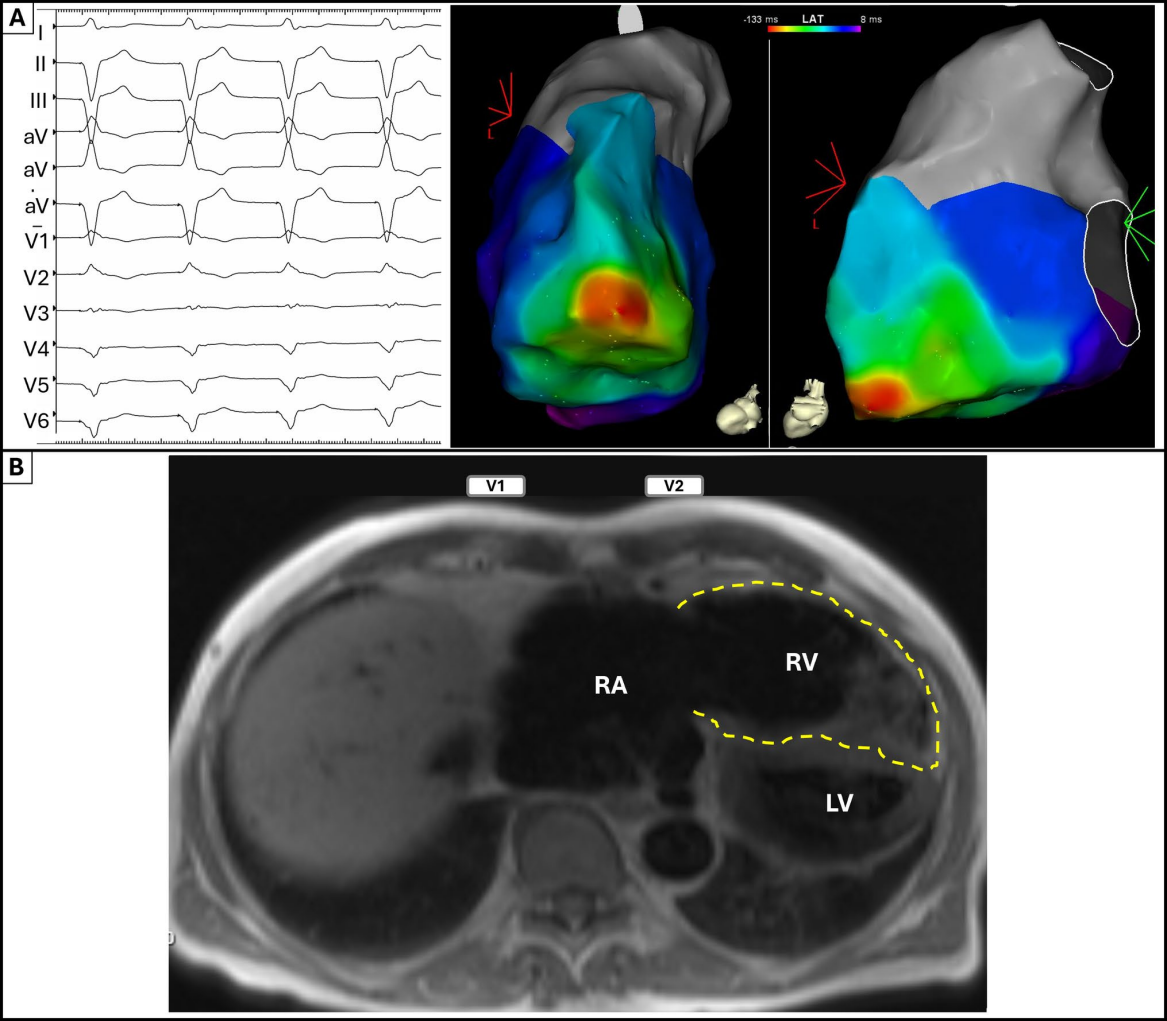
Unexpected right bundle branch block pattern with electrical stimulation from the right ventricular apex. **A** RV apical threshold pacing from an ICD lead during an electrophysiological procedure demonstrates RBBB morphology with a precordial QRS transition from positive to negative in V3. The electroanatomic map depicts the corresponding activation wavefront which is spreading from the RV apex towards the base. **B** The RV is delineated by the yellow dashed line and identifies a severely dilated RV, with its apex displaced towards the left side of the thorax and wrapping around the apex of the comparatively small left ventricle. Positioning of leads V1 and V2 is depicted schematically, illustrating their relative positions to the heart to improve understanding of the activation wavefront vectors that produce the characteristic QRS pattern

In the presented case, intracardiac defibrillator (ICD) threshold pacing from an RV apical ICD lead was performed during an electrophysiological study (Fig. 1A). The magnetic resonance imaging (MRI) revealed extreme dilatation of the RV, with the RV apex extending beyond the left ventricular apex (Fig. 1B). A characteristic RBBB pattern with an early precordial transition was observed during pacing from the RV apex. Typically, this should raise suspicion for a misplaced lead but may occur in patients with dilated RV. The altered anatomical relationships, coupled with delayed activation, result in a delayed activation of the basal RV, producing an RBBB pattern despite the pacing originating from the RV apex. Electroanatomical mapping demonstrated RV activation spreading from the apex of the RV with its base and infundibulum activated more than 130 ms later, compatible with the RBBB pattern (Fig. 1A). Similarly, VT exiting from the RV apex in ARVC patients may also exhibit an RBBB pattern and thus does not necessarily indicate an origin from the left ventricle [3] . This report for the first time illustrates the correlation of the spreading activation wavefront on an electroanatomical activation map with this characteristic ECG pattern, providing insights into the underlying mechanisms.

Understanding this ECG phenomenon is crucial for interpreting RV pacing morphologies and localizing VT in ARVC patients, which may affect procedural planning and ultimately the success of an ablation procedure.
